# Observations on the Medical and Surgical Agency of the Air-Pump

**Published:** 1837-04

**Authors:** 


Art. VIII.—*Observations on the Medical and Surgical Agency of the Air-Pump.* By Sir James Murray, m.d.—*Dublin,* 1836. 8vo. pp. 63.

In a preceding article in the present number, we animadverted on the dangerous facility afforded by our numerous Societies for the publication of memoirs on subjects not sufficiently considered by the authors, and the necessity of greater strictness on the part of those intrusted with the management of the literary department of such societies. We are sorry to say that the present essay, which was originally read at the meeting of the British Association in 1835, or at least the substance of it, affords another proof of this necessity. Its object is to show that the partial removal and the increase of the pressure of the atmospheric air on various parts of the body, must be remedial agents of great utility. The rarefaction of the air surrounding the body and limbs is accomplished by means of a tin bath with an aperture for the head, around which is fitted an air-tight cloth to surround the neck: it is furnished with an exhausting syringe, and a mercurial gauge to measure the degree of rarefaction. An apparatus has also been adapted to inclose parts of the body only, as single limbs; but no descriptions are given, the reader being referred to the instrument maker. The removal of one pound weight of pressure from every square inch is the utmost required when the whole body below the neck is subjected to the operation; when it is applied topically, two or three pounds of pressure may be withdrawn from every square inch; but there is seldom occasion to proceed to this extent. The effect produced is congestion of the cutaneous surface and perspiration; when one limb is operated on, its size is for the time considerably increased. The greater part of Sir James Murray's pamphlet is devoted to a discussion of the diseases in which it is applicable as an adjuvant to other means. These are "all simple fevers, especially at the commencement," p. 10; "the cold stage of intermittents," p. 11; "all the shades and colours of cutaneous disorders depicted by Willan and Bateman," p. 12; "inflammation of the brain, lungs, pleura, heart, stomach, liver, or any other internal organ," p. 13; "simple congestive apoplexy," p. 14; "cases of paralysis depending on surcharge of the vessels of the brain or spinal marrow, and where coma, stupor, embarrassment of speech, distorted vision, intolerance of light or sound, and loss of memory, arose from the same causes," p. 19; "in preventing tubercular cachexia," p. 21; in "active and passive congestion" of the mucous membrane of the stomach and bowels," p. 22; in those complaints called nervous which "depend on various congestions, chronic enlargements, fulness, and sometimes sub-inflammatory states of parts of the brain, spinal marrow, or their investments," p. 23; and "in certain kinds of ailments of the eye, ear, and other organs of sense, epilepsy, spasmodic affections, mania, chorea, and several of the neuralgia," p. 24; in severe burns "where sphacelation is threatened," p. 29; "in the treatment of some female complaints," p. 30; in "partial paralysis," p. 30; "in dissection wounds," p. 34; "bites of mad dogs," p. 35; "lumbar abscess when opened," p. 37; "drawing out piles," p. 37; "drawing the breasts to promote menstruation," p. 40; &c.!

That this extensive dry cupping does actually produce permanent relief in so many diseases is not so much attempted to be proved, as that, according to theory, it ought to do so. Sir J. Murray's theory appears to be, that as these diseases depend on congestion of blood in some internal organ or structure, therefore artificially drawing the blood into the cutaneous vessels, must diminish the internal congestion and relieve the disease. He makes it a purely mechanical process, and thus contrasts it with derivation produced by medicinal revulsives, as diaphoretics, frictions, blisters, &c. "This is the great line of distinction between derivation of the fluids to the surface by suction, and its propulsion by the usual medicinal revulsives; that the former method acts in making the capillaries passive conductors of increased circulation, but the latter operate by rendering the vessels more *active*, aggravating acute cases and adding to their over-excitement." (P. 17.)

If we are to agree with this theory, it will be difficult to conceive that suction can exert much influence on any permanent inflammation. There are, however, proofs that the capillaries are not mere passive tubes in this process of rarefaction, for the part becomes red, painful, hot, and covered with perspiration, and the warmth and redness continue a considerable time after the pressure is restored. The want of permanency of this excitement seems to be the only theoretical reason against its general utility as a means of counter-irritation or derivation, and we suspect that dry cupping has gone out of fashion from the slight effect of so temporary a remedy over fixed diseases.

But it is not only, (argues Sir James,) that removal of atmospheric pressure relieves on the principle of dry cupping. When the whole body, except the head, is subjected to it, there will be an increased facility in expanding the chest, for the removal of the pressure of the atmosphere from the outside of the thorax allows the pressure within to dilate the chest more readily, and as the contractile power of the muscles proceeds without interruption in a partial vacuum, the expansion of the chest in inspiration is made easier without any hindrance to the due contraction in expiration. Hence, in all cases where "there is debility or torpor of the respiratory nerves and muscles," (p. 8,) this easier acquisition of pulmonary capacity is an important benefit.

In the absence of cases (and none are given,) it is not easy clearly to understand the particular kind of diseases indicated; for, theoretically, expiration should appear to be rendered more difficult by the removal of part of the atmospheric pressure externally. In ordinary circumstances, the external pressure of the air being equal to the internal pressure, the respective forces are balanced. But if the air is pressing into the lungs with its natural weight of 15lbs. on every square inch, and that on the external surface with only 14lbs., (1lb. being removed by the rarefying apparatus,) a greater contractile power is necessary to expel it. The muscles necessary for expiration should therefore neither be weak nor torpid.

In the observations made on compression of the air, the descriptions of the mode of effecting it are so scanty and imperfect, that it is difficult for one who has not seen the actual application of the process to understand it. Much however is promised: it is to supersede the diachylon strapping of Velpeau in burns, to "regulate graduated compression upon angular or irregular joints or surfaces, and thus arrest hemorrhage," p. 49; to supersede bandages in fractures, and render "vascular, turgid, and inflamed ulcers" less active: in hernia humoralis, it is to relieve the pain and inflammation, and it is to dissipate tumours, and push up all kinds of protrusions, and press into the skin various medicaments. When a lady is unable "to nurse," or her child is dead, compression on the mammæ is to prevent the secretion of milk; as, on the other hand, when there is a paucity of milk, by means of rarefaction, ladies "may have the supply required." (P. 39.)

If such compression will actually produce these effects and can be readily employed, it will be a valuable addition to our stock of remedies; and, as Sir James Murray describes himself as "nervously anxious" that it should be understood, and appears to be fully convinced of its value, we are sorry that he has not given his observations that form which would have tended to produce a similar conviction in the minds of his readers.

Had he given a careful history of the cases falling under his own observation, with the necessary details of the length of time the rarefaction or compression was employed, the immediate results and their ultimate effects, even although these cases might have been but few, he would have done more to accredit the practice than can possibly be accomplished by the mere expression, however loud, of his own conviction of the value of his discovery and of its being applicable to almost the whole circle of diseases. That he has not done so we regret the more, from believing that the remedy in a more limited number of diseases is likely to be beneficial. The proposal to dry-cup the breasts in amenorrhœa, in order sympathetically to promote the action of the uterus, is worth attending to. If successful, it is a proof too that the beneficial effects of suction depend on the irritation of the mammary vessels, and not merely in dilating mechanically the passive capillaries; for the fact, that by drawing a certain quantity of blood into an organ, a larger quantity is determined to a distant part, can never be explained by mechanics. The value of rarefaction where a mechanical effect only is required, as in the reduction of hernia, has lately been proved by a large number of successful cases, (vide No. II. of this Journal, p. 586,) by Dr. Koehler of Warsaw. In justice to Sir J. Murray, it should be stated, that he had suggested the remedy previously to the appearance of Dr. Koehler's paper.

